# Angiography-derived fractional flow reserve- vs usual care-guided percutaneous coronary intervention: interim analysis of the PIONEER IV trial

**DOI:** 10.1093/eurheartj/ehag174

**Published:** 2026-03-17

**Authors:** Patrick W Serruys, Tsung-Ying Tsai, Joanna J Wykrzykowska, Faisal Sharif, Liesbeth Rosseel, Mohammad Alkhalil, Nick Curzen, Vincent Floré, Edouard Benit, Yoann Bataille, Mathieu Pankert, Clemens von Birgelen, Kenneth De Wilder, Valeria Paradies, Giovanni Amoroso, Lionel Mangin, Manel Sabate, Samer Somi, Adel Aminian, Sjoerd Hofma, Ignacio J Amat Santos, Victor Alfonso Jimenez Diaz, Gilles Barone-Rochette, Anthony Mathur, Jeroen Sonck, Julien Adjedj, Jacques Monsegu, Julien Lemoine, Michael Angioi, Carlos González Juanatey, Amer Zabalawi, Nozomu Kanehama, Akihiro Tobe, Kotaro Miyashita, Adrian Bednarek, Asahi Oshima, Albert Chinhenzva, Kanika Wadhwa, Scot Garg, Andreas Baumbach, Peter Smits, Yoshinobu Onuma

**Affiliations:** CORRIB Research Centre for Advanced Imaging and Core Laboratory, University of Galway, University Road, Galway H91 TK33, Ireland; CORRIB Research Centre for Advanced Imaging and Core Laboratory, University of Galway, University Road, Galway H91 TK33, Ireland; Department of Cardiology, University Medical Center Groningen, Groningen, the Netherlands; Department of Cardiology, University of Galway, Galway, Ireland; SFI infrastructure Funding, University of Galway, Galway, Ireland; Department of Cardiology, Algemeen Stedelijk Ziekenhuis, Aalst, Belgium; Cardiothoracic Centre, Freeman Hospital, Newcastle-upon-Tyne, UK; Translational and Clinical Research Institute, Newcastle University, Newcastle-upon-Tyne, UK; Faculty of Medicine, University of Southampton, Southampton, UK; Department of Cardiology, University Hospital, Southampton NHS Trust, Southampton, UK; Department of Cardiology, AZ Maria Middelares, Ghent, Belgium; Department of Cardiology, Jessa Hospital, Hasselt, Belgium; Department of Cardiology, Jessa Hospital, Hasselt, Belgium; Service de cardiologie, Centre Hospitalier Avignon, Avignon, France; Department of Cardiology, Thoraxcentrum Twente, Medisch Spectrum Twente, Enschede, the Netherlands; Health Technology and Services Research, Faculty of Behavioural, Management and Social Sciences, Technical Medical Centre, University of Twente, Enschede, the Netherlands; Heart Centre, Imelda Hospital Bonheiden, Bonheiden, Belgium; Department of Cardiology, Maasstad Hospital, Rotterdam, the Netherlands; Department of Cardiology, OLVG, Amsterdam, the Netherlands; Cardiology Department, Hospital Annecy-Genevois, Metz-Tessy, France; Interventional Cardiology Department, Cardiovascular Institute, University Hospital Clinic, IDIBAPS, Barcelona, Spain; Interventional Cardiology Department, Centro de Investigación Biomédica en red—Enfermedades Cardiovasculares (CIBERCV), Madrid, Spain; Department of Cardiology, Haga Hospital, The Hague, The Netherlands; Division of Cardiology, Centre Hospitalier Universitaire de Charleroi, Charleroi, Belgium; Department of Cardiology, FrisiusMC, Leeuwarden, the Netherlands; Interventional Cardiology Department, Centro de Investigación Biomédica en red—Enfermedades Cardiovasculares (CIBERCV), Madrid, Spain; Cardiology Department, University Clínic Hospital of Valladolid, Valladoild, Spain; Cardiovascular Research Area, Instituto de Investigación Biosanitaria de Valladolid (IBioVALL), Valladolid, Spain; Cardiology Department, Hospital Álvaro Cunqueiro, University Hospital of Vigo, Vigo, Pontevedra, Spain; Cardiovascular Research Group, Galicia Sur Health Research Institute (IIS Galicia Sur), SERGAS-UVIGO, Vigo, Spain; Department of Cardiology, University Hospital Grenoble France, Grenoble, France; Clinic of Cardiology, Cardiovascular and Thoracic Department, University Grenoble Alpes, INSERM, CHU Grenoble Alpes, Grenoble, France; French Alliance Clinical Trial, French Clinical Research Infrastructure Network, Paris, France; Barts Heart Centre, Barts Health NHS Trust, London, UK; Centre for Cardiovascular Medicine and Devices, William Harvey Research Institute, Queen Mary University of London, London, UK; NIHR Barts Biomedical Research Centre, Queen Mary University of London, Charterhouse Square, London, UK; Cardiovascular Center Aalst, AZorg Hospital Aalst, Aalst, Belgium; Arnault Tzanck Institute Cardiology Department, Arnault Tzanck Institute, Saint-Laurent-du-Var, France; Medipôle Private Hospital, GCV Cardiopôle, Grenoble, France; Department of Cardiology, Clinique Louis Pasteur, essey les Nancy, France; Department of Cardiology, Clinique Louis Pasteur, essey les Nancy, France; CardioHULA Research Group, Cardiology Department, Lucus Augusti University Hospital, Lugo, Spain; Department of Cardiology, Centre Hospitalier de Saint-Brieuc, 10 rue Marcel Proust, Saint-Brieuc 22000, France; CORRIB Research Centre for Advanced Imaging and Core Laboratory, University of Galway, University Road, Galway H91 TK33, Ireland; CORRIB Research Centre for Advanced Imaging and Core Laboratory, University of Galway, University Road, Galway H91 TK33, Ireland; CORRIB Research Centre for Advanced Imaging and Core Laboratory, University of Galway, University Road, Galway H91 TK33, Ireland; CORRIB Research Centre for Advanced Imaging and Core Laboratory, University of Galway, University Road, Galway H91 TK33, Ireland; First Department of Cardiology, Medical University of Warsaw, Warsaw, Poland; CORRIB Research Centre for Advanced Imaging and Core Laboratory, University of Galway, University Road, Galway H91 TK33, Ireland; CORRIB Research Centre for Advanced Imaging and Core Laboratory, University of Galway, University Road, Galway H91 TK33, Ireland; CORRIB Research Centre for Advanced Imaging and Core Laboratory, University of Galway, University Road, Galway H91 TK33, Ireland; Department of Cardiology, Royal Blackburn Hospital, Blackburn, UK; School of Medicine, University of Lancashire, Preston, UK; Department of Cardiology, Barts Heart Centre, Barts Health NHS Trust, London, UK; Centre for Cardiovascular Medicine and Devices, William Harvey Research Institute, Queen Mary University London, London, UK; Centre for Cardiovascular Medicine and Devices, William Harvey Research Institute, Queen Mary University London, London, UK; Department of Cardiology, Maasstad Ziekenhuis, Rotterdam, the Netherlands; CORRIB Research Centre for Advanced Imaging and Core Laboratory, University of Galway, University Road, Galway H91 TK33, Ireland

**Keywords:** Quantitative flow ratio guidance, Usual care guidance, Non-inferiority trial, All comers percutaneous coronary intervention, Healing-Targeted Supreme sirolimus-eluting stent, Ticagrelor monotherapy

Quantitative flow ratio (QFR), an angiography-based method for estimating fractional flow reserve (FFR) without pressure wire, can be integrated into percutaneous coronary intervention (PCI) workflows to guide lesion treatment selection and stent optimization.^1^ The FAVOR III China trial demonstrated superior clinical outcomes with QFR vs visual angiographic guidance, while the FAVOR III Europe trial failed to demonstrate clinical non-inferiority of QFR-guided PCI to FFR-guided PCI, raising concerns with increased rate of spontaneous myocardial infarction (MI), potentially impacting the Class I(B) guideline endorsement.^2–4^ To address these safety concerns, the PIONEER IV steering committee, consultatively advised by the Data Safety Monitoring Board (DSMB), decided to report an interim analysis of the first 1270 patients (60%) in the trial to offer timely insights for the community.

PIONEER IV (NCT04923191) is a multicentre, 1:1 randomized, open-label, all-comer, non-inferiority trial comparing clinical outcomes of PCI guided by QFR vs usual care, enrolling patients irrespective of clinical presentation or lesion complexity with unrestricted use in both arms of the Healing-Targeted Supreme sirolimus-eluting stent (HT Supreme, SINOMED, Tianjin, China), followed by a 1-month dual-antiplatelet therapy and ticagrelor monotherapy up to 1 year.^5^ In the QFR arm, lesions were functionally assessed by investigators with QFR, with stenting indicated in lesions with a QFR ≤0.80. Post-stenting, QFR was repeated in the stented vessel(s), with post-dilatation or additional stenting recommended if vessel QFR <0.91, or if the delta QFR (across the stent) was >0.05, with the use of intravascular imaging at investigator’s discretion. Usual care PCI was performed according to standard clinical practice, with pressure wire, angiographic physiology, and intravascular imaging use, left to the investigator's discretion.^2^

The primary non-inferiority endpoint is 12-month patient-oriented composite endpoint (POCE) of all-cause death, any stroke, any MI, or any Academic Research Consortium-2 clinically and physiologically driven revascularization with a non-inferiority margin of 3.2%.^6^

Clinical events were adjudicated by blinded clinical event committee and angiographic Syntax Score was assessed by an imaging corelab (CORRIB CORELAB, Galway, Ireland).^7^ MI was defined using the SCAI consensus for peri-procedure MI, and the Fourth Universal Definition of MI >48 h after the index procedure.^8^ Secondary endpoints included the individual components of POCE and bleeding academic research consortium (BARC) bleedings.^6,9^

The protocol was amended to accommodate an interim analysis with a modest reduction in the sample size from 2540 to 2130 patients lowering the statistical power from 90% to 85%. Considering the additional look, a one-sided *P*-value threshold of .035 was applied, based on the Lan-DeMets boundaries with Pocock alpha spending functions.^10^ Event rate was estimated by the Kaplan–Meier method. The primary analysis was performed in the intention-to-treat population.

Among the first 1270 patients, 631 were randomized to the QFR arm and 639 to the usual care arm, with 1-year follow-up data available for 622 (98.6%) patients and 618 (96.7%) patients in the QFR and the usual care arms, respectively, while vital status was available for all patients (*Figure 1*). The median age was 66.0 years (IQR 59.0–74.0), with 304 (23.9%) female participants, 405 (31.8%) presenting with ACS, 298 (23.5%) having diabetes mellitus and 258 (20.3%) classified as high bleeding risk. Mean anatomic SYNTAX scores in the QFR arm was 9.7 (6.9) vs 9.6 (6.8) in the usual care arm.

**Figure 1:**
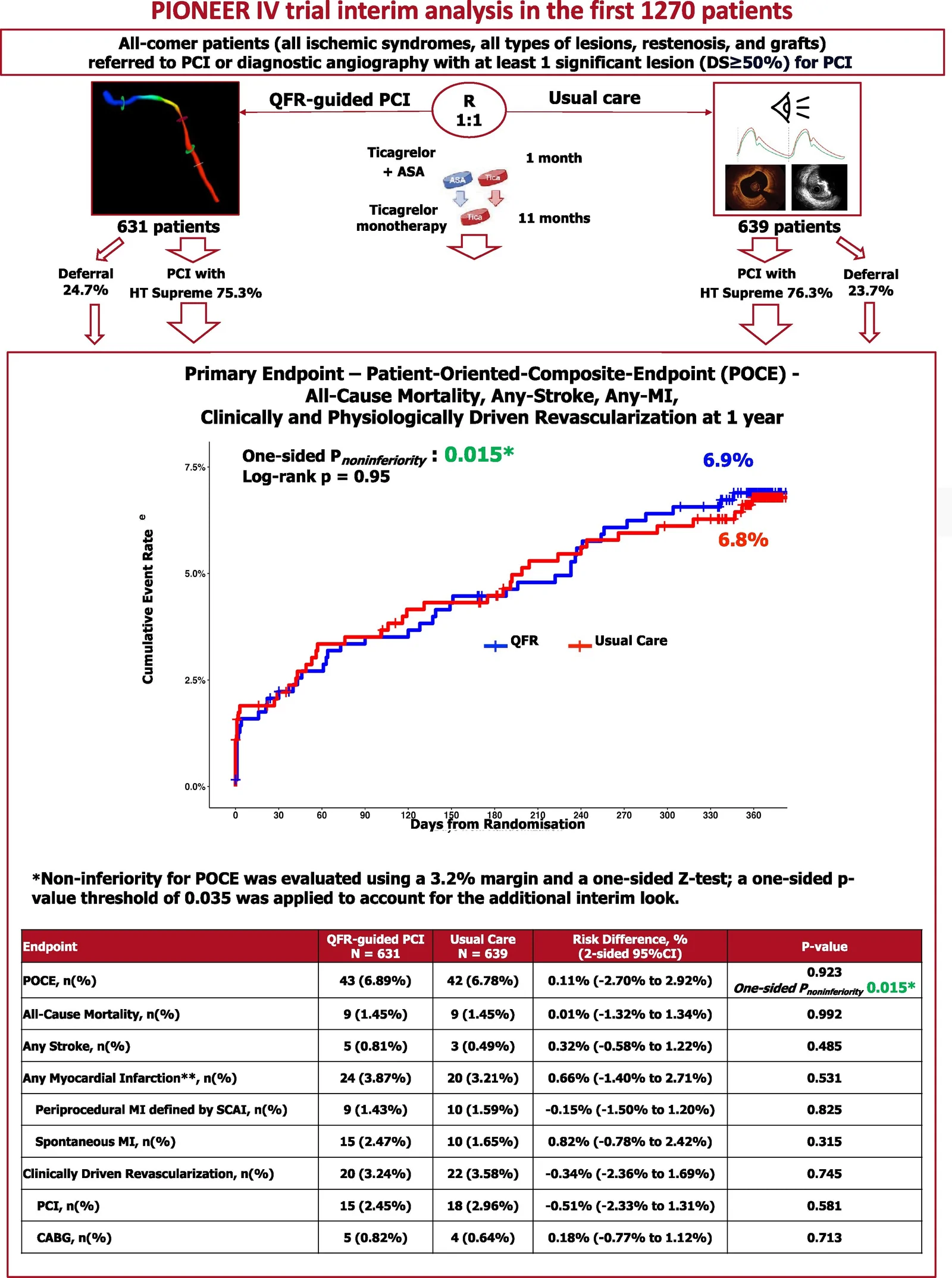
(Top) Trial design of the PIONEER IV study, a multicentre, open-label, randomized control trial comparing angiography-derived fractional flow reserve (QFR)-guided PCI vs usual care in an all-comer population. Patients received the Healing-Targeted Supreme sirolimus-eluting stent followed by 1 month of dual antiplatelet therapy and ticagrelor monotherapy up to 1 year. (Bottom) Kaplan–Meier cumulative event curves and event rates for the primary endpoint, the patient-oriented composite endpoint (POCE), at 1 year. The table details the individual components of POCE and key secondary endpoints, showing risk differences and *P*-values. *Non-inferiority for POCE was assessed using a one-sided *Z*-test with a threshold of 0.035. **Spontaneous Myocardial Infarction defined according to the Fourth Universal Definition while periprocedural MI defined according to the SCAI definition. ASA, acetylsalicylic acid; CABG, coronary artery bypass grafting; MI, myocardial infarction; PCI, percutaneous coronary intervention.

In the QFR arm, QFR was performed by investigators in 883 (90.8%) lesions and QFR guidance was followed in 862 (97.6%) lesions. In the usual care arm, pressure-wire-derived or angiography-derived physiological assessments in the cath lab were performed in 395 (41.0%) of the total 963 study lesions.

Overall, a similar proportion of lesions in both arms were deferred (QFR *n* = 343/973, 35.3% vs Usual care *n* = 319/963, 33.1%, *P* = .368).

The mean number of implanted stents and the total stent length per patient were 1.6 stents and 34.0 mm in the QFR arm and 1.6 stents and 34.8 mm in the usual care arm. Intravascular imaging was used in 4.6% and 5.3% of the QFR and usual care patients, respectively. Post-PCI physiology was assessed in 76.5% of the QFR arm patients, with 17.9% (56/313) having an angio-FFR < 0.91, leading to additional treatment in 19.6% (11/56) of cases. After PCI, patients who were not on anticoagulant therapy were on DAPT at 1 month, with P2Y12 monotherapy used in 83.0% at 6 months.

The 1-year POCE rates were 6.9% in the QFR arm and 6.8% in the usual care arm. With a risk difference of 0.11% (95% CI: −2.70% to 2.92%, one-sided *P*_noninferiority_ = .015, *Figure 1*), non-inferiority was declared. The individual components of POCE were similar between the two arms. Importantly, there were no between-group differences in the rates of all-MI, periprocedural MI or spontaneous MI. Furthermore, only one MI and two revascularizations occurring in deferred vessels. One acute definite stent thrombosis was observed in the usual care arm, and one late probable stent thrombosis was observed in the QFR arm. The BARC 3 or 5 bleeding rates were 1.8% and 1.3% in the QFR and usual care arms, respectively (*P* = .482).

The favourable outcomes relative to FAVOR III Europe likely reflect key design differences: PIONEER IV compared a QFR-guided strategy with usual care, whereas FAVOR III Europe mandated systematic FFR guidance. Consequently, PIONEER IV occupies an intermediate position on the ‘physiology spectrum’, with 41% invasive physiology use in the control arm, contrasting with the 100% FFR use in FAVOR III Europe and 0% in FAVOR III China. Furthermore, PIONEER IV mandated post-PCI QFR optimization. This strategy was found to be impactful in the FLAVOUR II trial, although that trial utilized a different technology (μFR) compared with the QFR used in PIONEER IV. Finally, rigorous site credentialing and centralized oversight ensured high protocol adherence (97.6%). Consequently, no alarming safety signals regarding spontaneous MI were observed. Limitations include the interim nature of the analysis with a limited statistical power and the open-label design.

In conclusion, QFR-guided PCI was non-inferior to usual care with no alarming safety signal in the interim analysis of the PIONEER IV trial. These results must be confirmed in the whole population.

## Permission information

The authors declare that all illustrations and figures in the manuscript are entirely original and do not require reprint permission.

## Declarations

### Data Availability

The study protocol and statistical analysis plan are available in the supplementary material. Other data, including study participant data and informed consent, will not be shared.

### Pre-registered Clinical Trial Number

The pre-registered clinical trial number is NCT04923191.
